# Are the opportunities to prevent alcohol related liver deaths in the UK in primary or secondary care? A retrospective clinical review and prospective interview study

**DOI:** 10.1186/1747-597X-1-16

**Published:** 2006-06-15

**Authors:** Clare Verrill, Stewart Smith, Nick Sheron

**Affiliations:** 1 Histopathology Department, Southampton University Hospitals NHS Trust, Southampton General Hospital, Tremona Road, Southampton. SO16 6YD, UK; 2 Division of Infection, Inflammation and Repair, Southampton University Hospitals NHS Trust, Southampton General Hospital, Tremona Road, Southampton. SO16 6YD, UK

## Abstract

**Background:**

Deaths from liver cirrhosis have increased at least 8 fold since the 1970's in the UK and further increases are anticipated, whereas in the rest of Europe liver deaths are decreasing. In the UK, we urgently need strategies to detect those who misuse alcohol and are at risk of developing alcoholic liver disease before they get to that point. One potential strategy is to screen admissions to hospital with alcohol related conditions for evidence of alcohol misuse.

Surprisingly, there has been no research into the important question of where the opportunities are to detect those who misuse alcohol – primary or secondary care. We attempted to answer this firstly by conducting a retrospective analysis of the medical notes of 94 patients diagnosed with alcohol induced liver cirrhosis between 1^st ^January 1995 and 31^st ^December 2000 at Southampton General Hospital with the purpose of identifying admissions to hospital prior to a diagnosis of alcoholic liver disease. In the second part of the study, we interviewed patients with alcoholic liver disease about their contact with health services.

**Results:**

Before diagnosis of alcoholic liver disease, 33% (31/94) of the patients had had an admission to hospital for an alcohol related condition. There was a mean of 7 years and 1 month (SD 6 years 3 months) between the first alcohol-related admission and presentation with alcoholic liver disease (in those who had had admissions). The commonest reason for alcohol related admission was falls/fractures/injuries, followed by non-variceal gastro-intestinal bleeds. Patients with alcoholic liver disease who were interviewed had seen their General Practitioner on average at least 2 times per year.

**Conclusion:**

Most patients who develop alcohol-induced cirrhosis do not have an admission to hospital with an alcohol related condition before developing alcoholic liver disease. Therefore, if we screen patients admitted to hospital with alcohol related conditions for evidence of alcohol misuse, we could potentially detect around a third of those at risk of developing cirrhosis. Although secondary care has an important role to play in detecting those at risk, the main opportunity for detection is in primary care.

## Background

Deaths from liver cirrhosis have increased at least 8 fold since the 1970's and further increases are anticipated whereas in the rest of Europe liver deaths are decreasing [1]. Rates of cirrhosis are directly influenced by alcohol consumption in a population and alcohol consumption doubled in the UK between 1960 and 2002 [2]. The UK Government Alcohol Strategy reported that alcohol causes at least 22,000 deaths and costs the UK £15 billion every year [3]. Data from the General Household Survey shows that 27% of UK men and 17% of women drink more than the government recommended maximum of 21 and 14 units/week [4]. Of these, 5% of men and 3% of women drink more than 50 or 35 units/week – the level where liver disease starts to become a major health risk. Many of these individuals will develop significant liver disease (alcoholic hepatitis/cirrhosis) – a clinical process which is usually entirely silent with no signs or symptoms [5,6] and the majority of these patients will not have established alcohol dependency [7]. Of those that present with a sudden variceal haemorrhage or decompensated liver disease with ascites, one third will die within a month, a further third will die within a few months and only one third will survive long term [8,9], figures which have not improved in the UK over the last 35 years [8]. With such high mortality figures when patients present for the first time with cirrhosis, the emphasis must be on detection and treatment of these individuals before they reach this point.

The Royal College of Physicians published a report in 2001 entitled 'Alcohol – Can the NHS (National Health Service) Afford it' [10] suggesting various strategies to combat the UK's growing alcohol problem. It recommended screening those who are admitted to hospital with an alcohol related condition, for example, head injuries or drug overdose for evidence of alcohol misuse. The best way of screening was thought to be some kind of questionnaire, for example, AUDIT (Alcohol Use Disorders Identification Test) which identifies hazardous drinkers. It also suggested that it may even be necessary to screen all admissions to hospital, whatever the reason. The UK Government subsequently published an 'Alcohol Harm Reduction Strategy for England' in 2004 [11] which was criticised for failing to address the health issues.

Brief interventions are an effective way to detect and treat those with hazardous drinking patterns and are usually used in primary care, although can be used in other settings, for example, Accident and Emergency Departments. They involve 5–10 minutes of advice given on an opportunistic basis when patients attend for other problems. Brief intervention works in half of subjects [12] and when liver disease is diagnosed at least half stop drinking. 90% of people visit their General Practitioner (GP) in a 5 year period [13] meaning that primary care potentially plays a pivotal role in detection and treatment of alcohol misusers.

So where are the opportunities to detect hazardous and harmful alcohol drinkers, in primary (General Practice) or secondary (hospital) care? This is the question we attempted to answer in this study firstly using a retrospective clinical review of patients with alcohol-induced liver cirrhosis and secondly by interviewing patients with alcoholic liver disease about their contact with primary and secondary care before being diagnosed with alcoholic liver disease. Surprisingly, we have not been able to find any previous studies addressing this very important question.

In this study we show that most patients who develop alcohol-induced liver cirrhosis do not have an admission to hospital for an alcohol related condition before developing alcoholic liver disease, but that they do have contact with primary care.

## Methods

Retrospective analysis of hospital records and a prospective interview study in patients presenting with alcohol related liver disease. Ethical approval was obtained for both parts from the local ethics committee (Part 1 – Isle of Wight, Portsmouth and South East Hampshire Local Ethics Committee ref no 06/Q1701/19, Part 2 – Southampton and South West Hampshire Ethics Committee (B) ref No 400/02/S/W) and conformed to the principles embodied in the Declaration of Helsinki.

### 1) Retrospective clinical review of patients with alcohol-induced liver cirrhosis

All patients who had a liver biopsy between 1^st ^January 1995 and 31^st ^December 2000 at Southampton General Hospital were identified from the pathology department computer system. The reports of these biopsies were reviewed and any containing a diagnosis of cirrhosis (including incipient or imminent cirrhosis) and a histological picture suggestive of alcohol were flagged up for the study (n = 109). It is our policy to make a histological diagnosis in patients with significant liver failure via a transjugular biopsy, but restricting the study population to biopsy proven cirrhosis would have excluded some subjects with less significant liver disease. This was a deliberate decision to ensure a homogeneous study population. The clinical history was reviewed to corroborate that there was a history of excess alcohol. Any without this history were excluded from the study on the basis that they were probably non-alcoholic fatty liver disease and any with any other form of significant liver co-morbidity eg hepatitis C were excluded, leaving 104 patients. All our patients undergo serological testing for viral hepatitis. The clinical notes were reviewed and complete data was obtained on 94 patients (in 4 the notes could not be found and in 6 only partial data could be obtained and therefore these patients were excluded from data analysis).

The socio-demographic and clinical characteristics of this group were as follows: 52 male, 42 female; mean age 49 years 2 months; 69 had ascites at the time of biopsy (25 did not); 45 had varices at the time of biopsy (24 did not and 25 were unknown); 10 were Childs-Pugh Grade A, 42 were Grade B, 40 were Grade C and 2 were unknown.

The date at which each patient was first identified as having alcoholic liver disease was recorded and this was defined as either presenting with alcoholic hepatitis or cirrhosis, or having a biopsy showing alcohol-induced cirrhosis. Any admissions to hospital prior to this diagnosis were identified and were further divided into incidental admissions (no apparent relation to alcohol, for example, elective operations) and alcohol related admissions (including drug overdose, non-variceal gastro-intestinal bleeds, pancreatitis, fractures, head injuries etc).

### 2) Interview study of patients with alcoholic liver disease

Patients with alcoholic liver disease were recruited from the liver wards and liver outpatient clinics at Southampton University Hospitals NHS Trust. 45 subjects were identified and interviewed as part of a wider study into alcohol. Part of this study used a locally developed questionnaire to determine contact with health care services regarding alcohol intake and alcohol related health problems. Specifically the questions asked were *'when was the first time, if ever, that you came to the attention of a health-care professional as a result of excessive alcohol intake?'*, *'how many times have you seen your GP in the last five years'*, *'have you ever been admitted to hospital/seen in Accident and Emergency for alcohol related problems in the past? If yes give details and provide number of days spent in hospital due to alcohol'*. Subjects were not considered suitable for interview if they were experiencing hepatic encephalopathy or severe withdrawal symptoms.

Data were recorded and analysed using Microsoft Excel and SPSS.

## Results

### 1) Retrospective clinical review of patients with alcohol-induced liver cirrhosis

Overall, 38 patients out of 94 total (40%) had had an admission to hospital for any reason before being diagnosed with alcoholic liver disease and the average number of admissions per subject (in those who had had an admission) was 2.8 (SD 2.1). This included 31 patients (33%) who had had an admission to hospital for a reason that might have been classed as alcohol related before being diagnosed with alcoholic liver disease and the average number of alcohol related admissions was 2.3 (SD 2.1). 67% had therefore had no admission to hospital for an alcohol related condition.

The mean length of time between the first admission to hospital for any reason (incidental or alcohol related) and being diagnosed with alcoholic liver disease was 10 years and 4 months (SD 7 years 0 months). The mean length of time between the first alcohol related admission to hospital and being diagnosed with alcoholic liver disease was 7 years and 1 month (SD 6 years 3 months). The reasons for alcohol related admissions are shown in table 1. 17% of patients (16/94) died within 3 months of their first presentation with alcoholic liver disease. Only 6 of these patients had had a prior admission to hospital with an alcohol related condition.

**Table 1:** Reasons for alcohol related admissions prior to developing alcoholic liver disease as determined from a retrospective analysis of medical notes in 94 patients with alcohol-induced cirrhosis (total number of admissions).

Reason for admission	Total number of admissions
Fall/fracture/injury (including head injury)	16
Gastro-intestinal bleed (non-variceal)	12
Drug overdose	11
Loss of consciousness/seizure	7
Abdominal pain due to eg gastritis, peptic ulcer disease	6
Alcohol overdose	4
Oral malignancy	2
Pneumonia	2
Confusion/Wernicke's encephalopathy	2
Oedema	1
TB	1

### 2) Interview study of patients with alcoholic liver disease

Patients presented after an average of 16 years of heavy drinking to health services. In response to the question *'how many times have you seen your GP in the last five years'*, those who were still drinking had seen their GP a mean of 9 times per year (16–44 age group) or 13 times per year (45–64 age group). Those who were abstinent had seen their GP a mean of 2 times per year (16–44 age group) or 6 times per year (45–64 age group).

In response to the question *'Have you ever been admitted to hospital/seen in Accident and Emergency for alcohol related problems in the past?' *there was a mean of 3 admittances/attendances per subject (range 0–17). Subjects had spent a mean of 23 days as an inpatient.

## Discussion

Retrospective clinical review of patients with alcohol-induced cirrhosis showed that before being diagnosed with alcoholic liver disease, most of the patients (67%) did not have an admission to hospital with an alcohol related condition during which they could have been screened for alcohol misuse. This therefore has implications for targeting of resources as most of these patients would need to have been detected in primary care. If there was an effective means to screen 100% of all admissions to hospital, then out of this cohort of patients a maximum of 40% could have been detected as hazardous drinkers. There was a mean of approximately 2 opportunities for detection ie admissions to secondary care in those who did present as such. Slightly more admissions were found in the interviews where the patients with alcoholic liver disease had attended a mean of 3 times per subject.

16 of the 94 patients died within 3 months of their first presentation with alcoholic liver disease. This is extremely important because these patients probably had no warning of the harm they were causing their liver and the first time they became aware of it, they had presented with a fatal condition. These patients had no chance to stop drinking and change the course of their illness because it was too late. If we can identify these patients and give them advice about their drinking before they develop cirrhosis, this at least gives them a chance to stop drinking. Simply telling someone they have alcoholic liver disease is enough to stop patients drinking or make them reduce their drinking to safe levels in just over half of patients in our experience (see below). Of the16 patients, only 6 had had a prior admission to hospital with an alcohol related condition, and therefore again, the main opportunity for detection and treatment would have been in primary care.

In those who presented to secondary care with an alcohol related condition, the mean time difference between the first presentation and detection of alcoholic liver disease was approximately 7 years. We know that around half of subjects respond positively to a brief invention [12] and similar results were seen in a cohort of heavy drinkers screened with gamma GT resulting in a significant reduction in liver morbidity [14]. Therefore if we can detect patients before they develop end stage liver disease we are likely to be able to reduce their alcohol consumption and thus avoid the development of cirrhosis in a substantial proportion. An audit recently carried out in Southampton showed that of 124 patients with alcoholic liver disease who survived one year after development of alcoholic liver disease, 54% were abstinent or drinking within liver safe levels (N. Sheron, personal communication). Patients were not given specific therapy for alcohol dependence, and thus figures represent the response of medical advice about their liver disease.

The first part of the study used only patients with cirrhosis who had had a biopsy, which was a deliberate attempt to study the patients with the most severe liver disease. All patients in Southampton with evidence of significant alcohol related liver disease are biopsied at some stage, so we felt this was a good way to detect patients with cirrhosis and that a large proportion would not be missed. Because only patients with alcohol-induced cirrhosis were selected, patients with non-cirrhotic but otherwise significant liver disease were not included because of this, for example those with alcoholic hepatitis and fibrosis and could lead to suggestion of selection bias. We feel that this actually strengthens our study as we used the patients who developed the most severe liver disease and most were not picked up by the health care system.

In the interview part of the study, patients had been drinking heavily for an average of 16 years before they presented to health services. In other words, there is a long 'latent' period in which we can detect patients who are drinking harmfully or hazardously during which we could reduce or stop their drinking. Most patients who develop alcoholic liver disease are not physically alcohol dependent [7].

A survey of drinking in adults conducted in the UK by the Office for National Statistics in 2004 showed that 11% of male drinkers and 8% of female drinkers had had a discussion with either their GP, someone else at the GP surgery, a doctor elsewhere or another medical person elsewhere about their drinking of alcohol in the last year [15]. Of the 11% of male drinkers who had this discussion, 9% of the discussions had been with their GP or someone else at the surgery, and only 2% had been with a doctor elsewhere or another medical person elsewhere and similar results had been obtained for women.

This study suggests that there is a place for detection of harmful and hazardous drinkers by screening admissions to secondary care, but we cannot rely on this alone as most patients would not be picked up this way. Primary care therefore probably has the biggest role to play in detection of alcohol misusers. The FAST test was specifically developed to detect hazardous drinking and not just dependency, it takes 13 seconds and has a sensitivity and specificity of around 90% [16]. The wider use of this test followed by appropriate stepped interventions along the lines of those used in Copenhagen have the potential to reduce liver deaths. Detailed strategies for detection and management of heavy drinkers in primary care have been previously published [17]. In order to detect alcohol misusers in General Practice, there needs to be screening for alcohol intake and liver disease, but until we do that, deaths will not reduce.

## Conclusion

We have increasing rates of liver cirrhosis in the UK, directly related to increasing alcohol consumption. Strategies to identify those at risk of developing alcoholic liver disease, including cirrhosis are urgently needed. Patients who misuse alcohol can be identified using questionnaires, which can be administered in primary or secondary care. The Royal College of Physicians have recommended as one strategy to screen admissions to hospital with alcohol related conditions for evidence of alcohol misuse.

In this study, we showed that most patients who develop alcohol-induced cirrhosis are not admitted to secondary care with an alcohol related condition (or any condition) before they develop alcoholic liver disease. Thus the opportunities to detect them as alcohol misusers in secondary care before they develop alcoholic liver disease are limited. Patients with alcoholic liver disease who were interviewed had on average at least 2 episodes of contact with primary care per year and 90% of people visit their General Practitioner in a 5 year period, meaning that primary care is ideally placed for detection of those who misuse alcohol.

In summary, there are opportunities to detect patients at risk of developing alcoholic liver disease in secondary care but this study suggests that the majority of detection needs to be done in primary care. In this study, patients had been drinking heavily for an average of 16 years before presenting to health services and therefore there is ample opportunity to detect and treat those at risk of developing alcoholic liver disease before they cause significant damage to their health.

## Competing interests

The author(s) declare they have no competing interests.

## Authors' contributions

CV carried out the retrospective clinical analysis part of the study and drafted the manuscript.

SS carried out the interview part of the study.

NS was the senior advisor on the study and helped draft the manuscript.

## References

[B1] Leon DA, McCambridge J (2006). Liver cirrhosis mortality rates in Britain from 1950–2002: an analysis of routine data. Lancet.

[B2] Academy of Medical Sciences (2004). Calling time; The nation's drinking as a major health issue.

[B3] Prime Ministers Strategy Unit (2003). Interim Analytical Report.

[B4] Social Survey Division of the Office for National Statistics (2001). General Household Survey.

[B5] Bruguera M, Bordas JM, Rodes J (1977). Comparison of clinical, biochemical and histological findings in 154 individuals chronically abusing alcohol. Arch Pathol Lab Med.

[B6] Sheron N, Dubras L (2005). Alcohol: who is at risk?. Practitioner.

[B7] Smith S, White J, Nelson C, Lavers J, Davies M, Sheron N (2006). Severe alcohol related liver disease and alcohol dependence. Alcohol and Alcoholism.

[B8] Gluud C (2005). Mortality from cirrhosis: lack of progress over the last 35 years. Gut.

[B9] Sheron N, O'Grady JG, Lake JR, Howdle PD (2000). Alcoholic liver disease. Comprehensive clinical hepatology Harcourt.

[B10] Royal College of Physicians (2001). Alcohol – can the NHS afford it?.

[B11] Prime Minister's Strategy Unit (2004). Alcohol harm reduction strategy for England.

[B12] Bertholet N, Daeppen JB, Wietlisbach V, Fleming M, Burnand B (2005). Reduction of alcohol consumption by brief alcohol intervention in primary care: systematic review and meta-analysis. Arch Intern Med.

[B13] Wallace P, Cutler S, Haines A (1988). Randomised controlled trial of general practice intervention in patients with excessive alcohol consumption. BMJ.

[B14] Petersson B, Kristenson H, Trell E, Hood B (1985). Screening and intervention for alcohol-related disease in middle-aged men: the Malmo Preventive Programme. Ciba Found Symp.

[B15] Office for National Statistics (2004). Drinking Behaviour and Knowledge in 2004.

[B16] Health Development Agency (2002). Manual for the FAST alcohol screening test.

[B17] De Silva AN, Sheron N (2005). Alcohol Related Liver Problems – Strategies for Primary Care. Clinical Focus Primary Care.

